# Exposing and exploiting host–parasite arms race clues in SARS-CoV-2: a principally new method for improved T cell immunogenicity prediction

**DOI:** 10.1093/biomethods/bpad011

**Published:** 2023-07-25

**Authors:** Jaroslav Flegr, Ivana Králová Lesná, Daniel Zahradník

**Affiliations:** Laboratory of Evolutionary Biology, Department of Philosophy and History of Science, Faculty of Science, Charles University, Prague, Czech Republic; Experimental Medicine Centre, Institute for Clinical and Experimental Medicine, Prague 140 21, Czech Republic; Department of Anesthesiology and Intensive Care of the 1st Faculty of Medicine, Charles University, Prague 169 02, Czech Republic; Laboratory of Evolutionary Biology, Department of Philosophy and History of Science, Faculty of Science, Charles University, Prague, Czech Republic; Department of Biological Risks, The Silva Tarouca Research Institute for Landscape and Ornamental Gardening, Průhonice, Czech Republic

**Keywords:** T cells, immunogenic epitopes, epitopes, peptide vocabulary, COVID, SARS-CoV-2, origin

## Abstract

Computational prediction of T cell epitopes is a crucial component in the development of novel vaccines. T cells in a healthy vertebrate host can recognize as non-self only those peptides that are present in the parasite’s proteins but absent in the host’s proteins. This principle enables us to determine the current and past host specificity of a parasite and to predict peptides capable of eliciting a T cell response. Building upon the detailed mapping of T cell clone specificity for Severe Acute Respiratory Syndrome Coronavirus 2 (SARS-CoV-2) antigens, we employed Monte Carlo tests to determine that empirically confirmed T cell-stimulating peptides have a significantly increased proportion of pentapeptides, hexapeptides and heptapeptides not found in the human proteome (*P* < 0.0001, Cohen’s *d* > 4.9). We observed a lower density of potential pentapeptide targets for T cell recognition in the spike protein from the human-adapted SARS-CoV-2 ancestor compared to 10 other SARS-CoV-2 proteins originating from the horseshoe bat-adapted ancestor. Our novel method for predicting T cell immunogenicity of SARS-CoV-2 peptides is four times more effective than previous approaches. We recommend utilizing our theory-based method where efficient empirically based algorithms are unavailable, such as in the development of certain veterinary vaccines, and combining it with empirical methods in other cases for optimal results.

## Introduction

The adaptive immune system of vertebrates discriminates between self and non-self antigens primarily by detecting peptides originating from non-self proteins. The detection of unknown molecules (or any previously unknown entities) might seem a challenging or even unsolvable problem, especially in comparison with the detection of known molecules or entities, but evolution found an elegant way of solving this principally tricky task. It is based on an interplay of T cells, Major Histocompatibility Complex (MHC) proteins, and antigen-presenting cells [1].

Almost all nucleated cells in vertebrates continuously digest a sample of all their endogenous proteins. A subset of the resulting peptides is presented on their surface for inspection by T cells. Specifically, these are the peptides that exhibit affinity to some variants of their MHC class I proteins. Similarly, professional antigen-presenting cells, such as dendritic cells or B-cells, digest a sample of all proteins which were transported to their interior by phagocytosis or endocytosis, and present on their surface a subsample of this sample, that is, peptides with affinity to some variant of their MHC class II proteins. Antigen-presenting cells cannot discriminate between self and non-self peptides: both types of peptides are therefore presented on their surface for inspection by T cells. Clones of T cells, each recognizing a different peptide—or a small group of peptides—kept by non-covalent bonds in the groove of MHC proteins, likewise cannot discriminate between self and non-self peptides. But during maturation, they pass through the thymus, where T cells with a strong enough affinity to any peptide attached to the MHC molecules (peptides originated from self-proteins) either die or differentiate into a specialized type of T cells—regulatory T cells [2–4]. Due to this combination of negative selection and elimination of T cells which bear receptors with insufficient affinity to any MHC–peptide complex, most mature cytotoxic and helper T cells patrolling in our bodies recognize only non-self peptides, that is, peptides that are not presented in the thymus.

In their coevolutionary arms race with hosts, parasitic organisms gradually eliminate all unnecessary pentapeptides from their peptide vocabulary. It has been demonstrated that a parasitic lifestyle affects the size of organisms’ pentapeptide vocabulary, that is, the number of different pentapeptides in their proteins, more strongly than the size or complexity of their proteome [5]. To avoid recognition by host’s T cells, parasites with a broad host specificity eliminate all unnecessary pentapeptides, that is, they reduce the size of their peptide vocabularies. Parasites characterized by a narrow host specificity substitute peptides not present in the proteins of their natural host species with those which are present there. In particular, they mutate these peptides, potential targets of T cell recognition, into peptides present in the peptide vocabulary of their host species. In viruses, bacteria, and parasitic protozoa, this evolutionary process can take place both within the entire metapopulation or in particular infrapopulations, that is, in the populations of parasites within individual hosts. The process can be therefore relatively rapid and can even affect the progress of a disease in an individual patient. The resulting peptide vocabulary mimicry could be partly responsible for the phenomenon of host specificity, that is, for the fact that most parasite species have a limited range of potential host species [6].

The similarity between pentapeptide and hexapeptide vocabularies can potentially indicate the species that serves as the natural host of a specific parasite species. This kind of analysis has recently shown that the natural host of the ancestor of Severe Acute Respiratory Syndrome Coronavirus 2 (SARS-CoV-2) was probably a horseshoe bat, while the natural host of the donor of its spike gene was human [7]. The same study also suggested that treeshrews were likely the most recent hosts of the virus from which SARS-CoV-2 sourced most of its protein-coding genes, while rats were probably the most recent hosts of the virus from which the spike gene originated.

In this study, we tested a critical prediction of the peptide vocabulary mimicry theory: we investigated whether the immunogenicity of peptides of viral origin can be predicted based on the content of pentapeptides and hexapeptides which are not present in their host’s peptide vocabularies. We took advantage of a recently published list of 734 peptides which elicited a specific immune response of cytotoxic or helper T cells isolated from 99 post-COVID patients [8]. Using *in silico* methods, we sought the intersecting points between the list of real (empirically identified) T cell response targets and a list of potential T cell recognition targets, that is, peptides present in SARS-CoV-2 but absent in the human proteome.

## Materials and methods

The proteomes (predicted sets of all proteins of a given organism) of *Homo sapiens* (F _000001405.39) and SARS-CoV-2, Wuhan variant (GCF_009858895.2), were downloaded from the NCBI GenBank database. We prepared the peptide vocabularies of humans and SARS-CoV-2 as previously described [7]. Unlike previous studies, we included in the analysis all proteins, encompassing paralogs. We first preprocessed the proteomes by filtering out all comments, annotations, and special codes (e.g. for unknown amino acids and gaps). Subsequently, we generated lists of all unique pentapeptides, hexapeptides, and heptapeptides present in the peptidomes of SARS-CoV-2 and humans (their pentapeptide, hexapeptide, and heptapeptides vocabularies) using the ImunDist 2.0 program [https://doi.org/10.6084/m9.figshare.17711474.v3]. This program, *in silico*, cuts the proteins into overlapping peptides of a desired length (e.g. pentapeptides) and records a list of unique peptides of that length in the proteome (assembling a peptide vocabulary). Using R, we prepared a list of penta-, hexa-, and heptapeptides present in SARS-CoV-2 proteins but absent in human proteins, representing potential targets for T cell recognition. From the paper by Tarke *et al.* [8], we extracted the lists of T cell response targets found in Supplementary Tables S5 and S8 [8].

**Figure 1:**
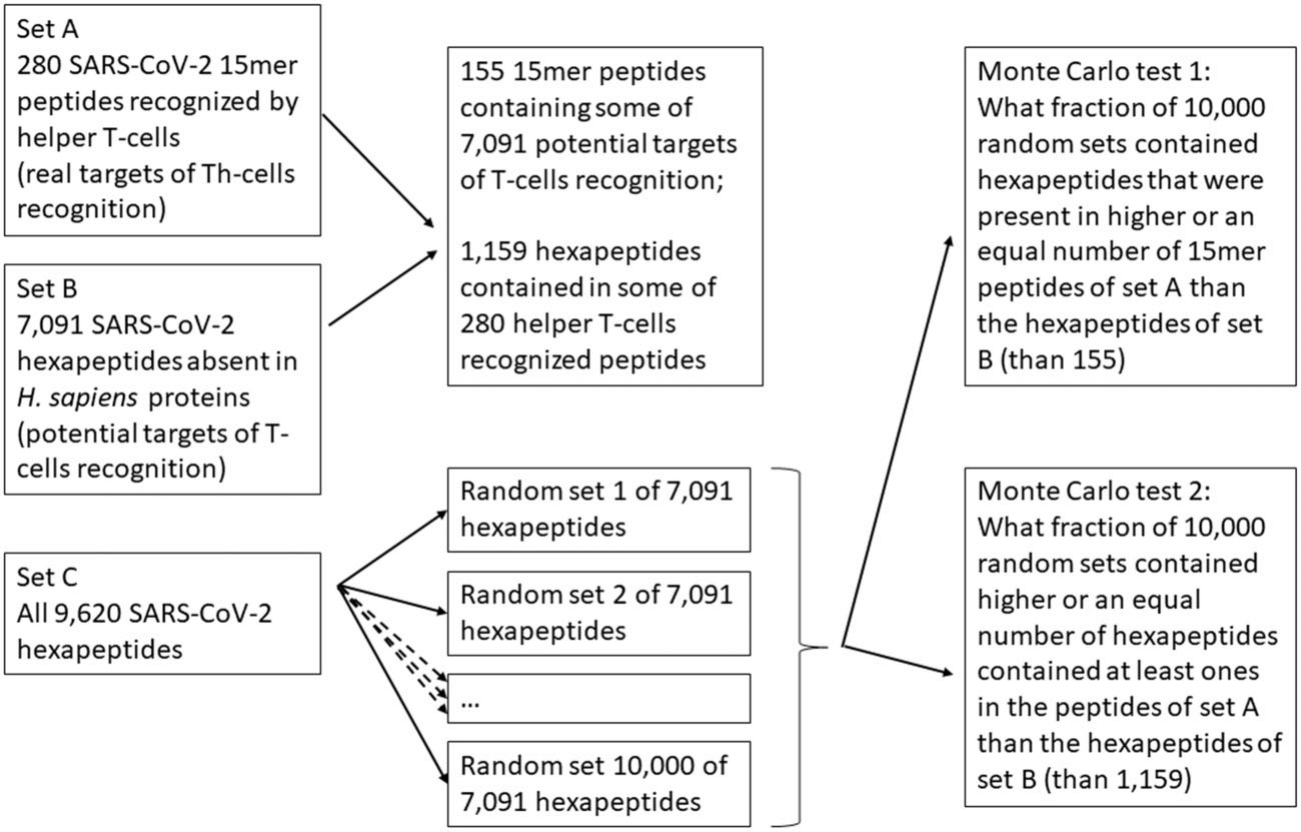
Schedule of Monte Carlo tests used in the present study. This schedule uses hexapeptides as an example. The same approach was applied to pentapeptides and heptapeptides.

To obtain a subset of genuine T cell response targets containing some of our potential targets for T cell recognition, the mcSortStrings 1.0 program [9] was used. Additionally, the mcPeptides 1.0 program [9] was employed to calculate the subset of potential T cell recognition targets contained within at least one genuine immune response target. Both programs (R scripts) were utilized to perform one-sided Monte Carlo tests (see Fig. 1).

Lists of potential targets for T cell recognition were generated for the entire SARS-CoV-2 proteome and separately for the spike protein, and the remaining 10 SARS-CoV-2 proteins were screened for T cell targets in the study [8] (products of genes M, N, nsp3, nsp4, nsp6, nsp12, nsp13, nsp16, ORF3a, ORF8). For both Monte Carlo tests, 10 000 sets of pseudo-targets were randomly selected from all overlapping peptides of a given length present in the SARS-CoV-2 proteome. These random sets were of the same size as the actual set of potential T cell recognition targets.

In the first Monte Carlo test (performed with mcSortStrings), significance was computed as the fraction of 10 000 random sets containing peptides present in an equal or higher number of genuine T cell response targets compared to peptides in the actual set of potential T cell recognition targets. In the second Monte Carlo test (performed with mcPeptides), significance was computed as the fraction of 10 000 random sets with an equal or higher number of peptides contained in the genuine T cell response targets than the peptides contained in the genuine set of potential T cell recognition targets. We performed post hoc tests specifically to investigate whether helper or cytotoxic T cells were responsible for the observed effect. As these were exploratory tests following our initial analysis, no correction for multiple testing was applied.

All computations, including Monte Carlo tests, were performed using the R programming language (version 2022.12.0+) and its standard base package [10]. The analysis was conducted within the IntelliJ IDEA integrated development environment (version 2021.3.3, Community Edition). The R scripts utilized in this study were developed, tested, and executed in the IntelliJ IDEA environment [11], taking advantage of its comprehensive toolset for code editing and debugging. The scripts and corresponding usage instructions are available at https://github.com/jflegr/peptides [9].

## Results

### How many potential targets of human T cell recognition are there in SARS-CoV-2 proteins?

**Figure 2:**
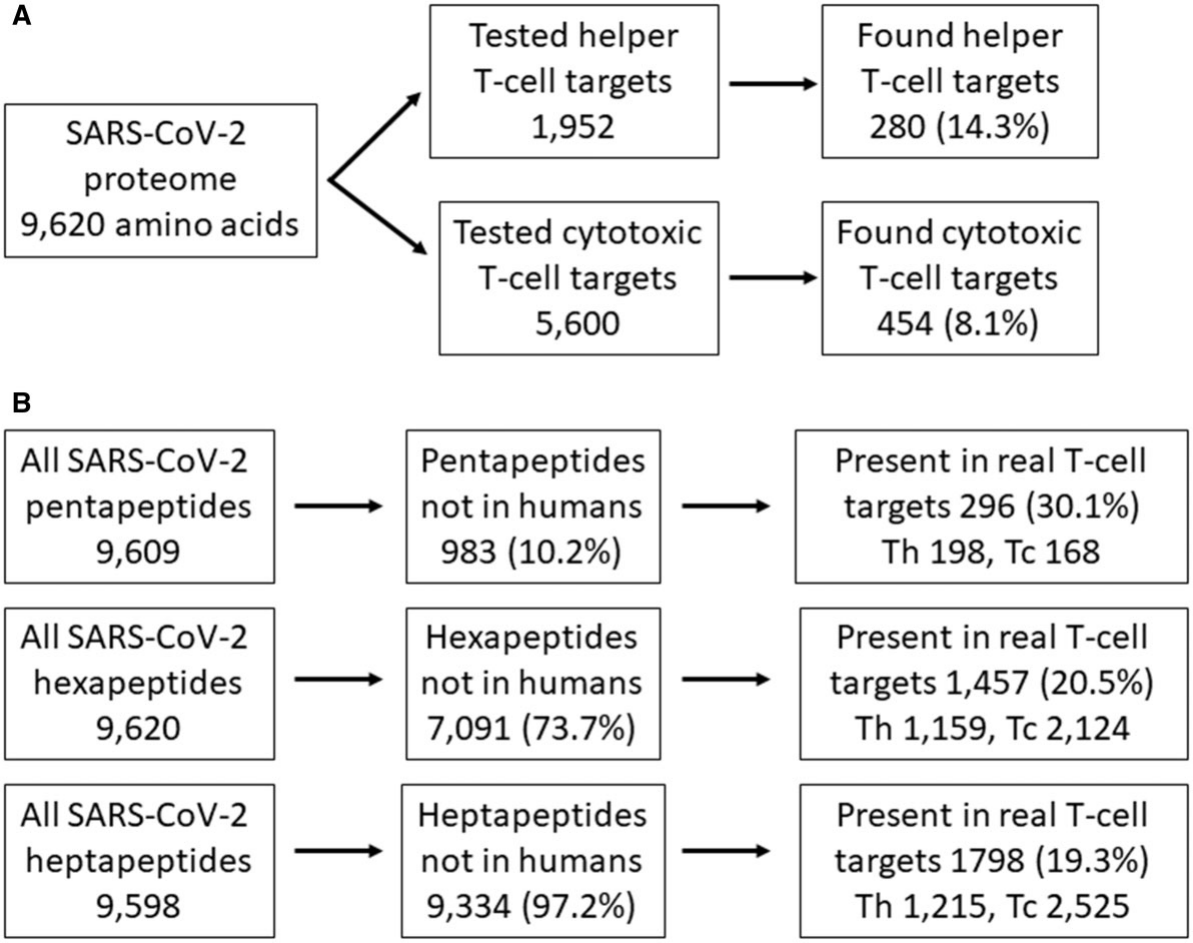
Classification and representation of targets of T cell response and potential targets of T cell recognition. (**A**) Offers a basic outline of genuine targets of helper and cytotoxic T cell response in empirically tested SARS-CoV-2 peptides [8]. (**B**) It shows a basic outline of potential targets of T cell recognition contained in peptides eliciting T cell response in all potential targets of T cell recognition and in all SARS-CoV-2 peptides.

We prepared penta-, hexa-, and heptapeptide vocabularies for humans and SARS-CoV-2 as well as lists of peptides present in the SARS-CoV-2 proteome but absent in the human proteome, that is, lists of potential targets of T cell recognition. The genetic distance between SARS-CoV-2 and humans is considerable, which is also why most viral peptides longer than six amino acids were absent in human proteins. Our analyses found that 983 (10.2%) of all 9609 pentapeptides, 7091 (73.7%) of all 9620 hexapeptides, and 9334 (97.2%) of all 9598 heptapeptides of SARS-CoV-2 were absent in human peptides and therefore could serve as potential targets of human T cell recognition (Fig. 2). The density of potential pentapeptide targets of T cell recognition was lower in the spike than in 10 other SARS-CoV-2 proteins (8.75% versus 11.07%, χ^2^ = 5.80, *P* = 0.016) [7]. No difference in the density of potential targets was observed for hexapeptides (7.43% versus 7.44%, χ^2^ = 0.01, *P* = 0.922) or heptapeptides (9.79% versus 9.72%, χ^2^ = 1.84, *P* = 0.175).

We calculated the sensitivity of the new method as the fraction of peptides identified by Tarke *et al.* as real targets of T cell response which contained at least one of our potential penta-, hexa-, and heptapeptide targets of T cell recognition. Then, we used a Monte Carlo test to estimate whether this fraction is larger than the equivalent fraction calculated for a case where potential targets of T cell recognition are selected from SARS-CoV-2 peptide vocabularies randomly (or, alternatively, where our method of recognition of T cell targets does not work).

**Figure 3:**
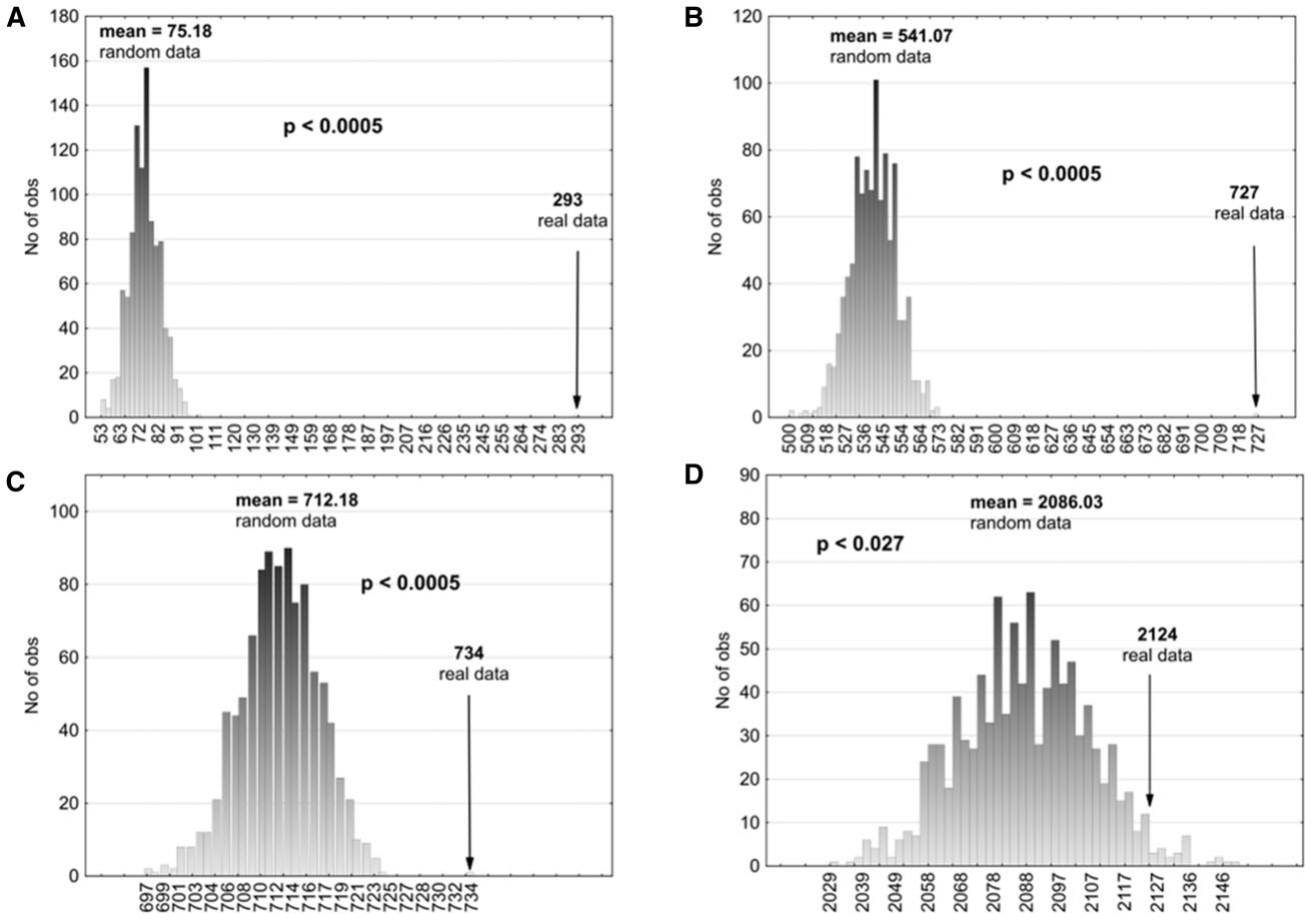
Results of the Monte Carlo test. (A–C) Figures display an overrepresentation of potential targets found in T cell response-eliciting peptides among all potential T cell recognition targets (penta-, hexa- and heptapeptides, respectively). (**D**) Illustrates an overrepresentation of T cell response-eliciting peptides containing potential T cell recognition targets (hexapeptides).

Tarke *et al.* tested 1952 15mer peptides covering whole SARS-CoV-2 proteomes and found 280 (14.3%) peptides which were, according to their criteria, the targets of actual helper T cell response (Fig. 2A). Using predictive algorithms, they found 5600 potential targets of response by cytotoxic T cells; in subsequent empirical essays, they found that 454 (8.11%) of them actually elicited a response by cytotoxic T cells. We found that 293 (39.9%) of all 734 targets of response by helper or cytotoxic T cells contained some of our 983 potential pentapeptides, 727 (99.0%) some of our 7091 potential hexapeptides, and 734 (100%) some of our 9334 heptapeptide targets of T cell recognition. Monte Carlo tests showed that the potential pentapeptide and hexapeptide T cell targets were strongly overrepresented in peptides which were recognized by helper T cells. When 983 pentapeptides and 7091 hexapeptides were selected 10 000 times randomly from all SARS-CoV-2 pentapeptides and hexapeptides, only, on average, 75.18 (10.2%) and 541.07 (73.7%) peptides eliciting T cell response contained some of the randomly selected pentapeptides and hexapeptides, respectively. The difference in representation of our potential targets of T cell recognition and the “pseudotargets” of randomly selected sets in peptides that really elicited T cell response was highly significant for both pentapeptides (39.9% versus 10.2%, *P* < 0.0001, Cohen’s *d* = 27.50) and hexapeptides (99.0% versus 73.7%, *P* < 0.0001, Cohen’s *d* = 16.2). In fact, pseudotargets of all of the 10 000 random sets of peptides were represented less frequently in peptides eliciting T cell response than in the genuine set of our potential targets of T cell recognition. Potential heptapeptide targets of recognition were present in all peptides eliciting a T cell response, while randomly selected pseudotargets were present only in 97.0% of such peptides (Fig. 3A–C). Again, the overrepresentation of genuine potential targets in peptides eliciting a T cell response was highly significant (100% versus 97.0%, *P* < 0.0001, Cohen’s *d* = 4.98). The effect size of all observed effects was very large; according to the widely used Cohen’s nomenclature, all effects with Cohen’s *d* > 0.8 are classified as large [12].

*Post hoc* analyses were performed separately for targets of response by helper and cytotoxic T cells which originated from the spike protein and the rest of the SARS-CoV-2 proteome (see Table 1). Analyses showed that the effect—that is, the overrepresentation of pentapeptide and hexapeptide potential targets of recognition in real targets of T cell response—was about twice stronger for helper T cells than for cytotoxic T cell targets, and it was weaker for the spike protein than for other proteins. The frequency of immunogenic peptides containing potential targets of T cell recognition was lower in the spike protein than in other SARS-CoV-2 proteins (see Table 1).

**Table 1:** Prevalence of potential targets of T cell recognition in peptides that elicited a T cell response

Proteins/vocabularies	No. targets	With genuine targets	With genuine targets (%)	With random targets	With random targets (%)	SD random targets	*P*	Cohen’s *d*
T cells, whole virus								
5-Peptides	734	293	39.9	75.18	10.2	7.92	0.000	27.50
6-Peptides	734	727	99.0	541.07	73.7	11.48	0.000	16.20
7-Peptides	734	734	100.0	712.18	97.0	4.38	0.000	4.98
Th cells, whole virus								
5-Peptides	280	155	55.4	28.72	10.3	5.04	0.000	25.04
6-Peptides	280	280	100.0	206.41	73.7	7.30	0.000	10.10
7-Peptides	280	280	100.0	271.65	97.0	2.78	0.000	3.01
Tc cells, whole virus								
5-Peptides	454	138	30.4	46.50	10.2	6.31	0.000	14.50
6-Peptides	454	447	98.5	334.65	73.7	9.24	0.000	12.16
7-Peptides	454	454	100.0	440.45	97.0	3.53	0.000	3.84
T cells, spike protein								
5-Peptides	247	86	34.8	21.63	8.8	3.96	0.000	16.26
6-Peptides	247	241	97.6	183.40	74.3	6.14	0.000	9.40
7-Peptides	247	247	100.0	241.76	97.9	2.04	0.002	2.58
Th cells, spike protein								
5-Peptides	92	46	50.0	8.02	8.7	2.63	0.000	14.44
6-Peptides	92	92	100.0	68.36	74.3	4.05	0.000	5.84
7-Peptides	92	92	100.0	90.03	97.9	1.35	0.135	1.46
Tc cells, spike protein								
5-Peptides	155	40	25.8	13.53	8.7	3.25	0.000	8.14
6-Peptides	155	149	96.1	115.16	74.3	5.13	0.000	6.60
7-Peptides	155	155	100.0	151.72	97.9	1.69	0.031	1.94
T cells, other proteins								
5-Peptides	487	207	42.5	53.84	11.1	6.62	0.000	23.13
6-Peptides	487	486	99.8	362.47	74.4	9.20	0.000	13.43
7-Peptides	487	487	100.0	473.34	97.2	3.50	0.000	3.94
Th cells, other proteins								
5-Peptides	188	109	58.0	20.83	11.1	4.22	0.000	20.90
6-Peptides	188	188	100.0	139.93	74.4	5.92	0.000	8.12
7-Peptides	188	188	100.0	182.73	97.2	2.25	0.004	2.34
Tc cells, other proteins								
5-Peptides	299	98	32.8	33.90	11.3	5.21	0.000	12.45
6-Peptides	299	298	99.7	222.54	74.4	7.26	0.000	10.40
7-Peptides	299	298	99.7	290.58	97.2	2.74	0.000	3.07

This table presents the total number of potential T cell recognition targets (Column 2, *No. targets*), the count of those actually present in peptides eliciting T cell responses (Column 3, *Within genuine*), their proportion among all potential T cell recognition targets (Column 4, *Within genuine %*), and the results of the Monte Carlo test. Specifically, the last five columns display the mean number of randomly selected pseudotargets present in peptides eliciting a T cell response (Column 5, *Within random*), their proportion within all pseudotargets (Column 6, *Within random %*), the standard deviation of the number of pseudotargets present in peptides eliciting a T cell response (Column 7, *SD random*), the significance of the one-sided Monte Carlo test (Column 8, *P*), and a Cohen's d indicating the effect size, representing the difference between columns 3 and 5 (Column 9, *Cohen's d*).

### What fraction of potential T cell targets were present in the known targets of T cell response?

We calculated the specificity of the new method as the fraction of our potential penta-, hexa-, and heptapeptide targets of T cell recognition that were present in peptides identified by Tarke *et al.* as real targets of T cell response. Tarke *et al.* found 280 15mer peptides recognized by Th cell and 454 peptides, 9–14 amino acids in length, that elicited a Tc-cell response (Fig. 2A). We found that 296 (30.1%) of the 983 identified potential pentapeptide targets, 2124 (30.0%) of the 7091 identified potential hexapeptide targets, and 2525 (27.1%) of the 9334 heptapeptide targets were present either among the empirically identified peptides stimulating the Th cells (pentapeptides: 198; hexapeptides: 1457; heptapeptides: 1798) or among Tc-cell epitopes (pentapeptides: 168, hexapeptides: 1159, heptapeptides: 1215), see Fig. 2B. Some of them, namely 70 pentapeptides, 492 hexapeptides, and 488 heptapeptides, were even present in both Th-cell and Tc-cell epitopes.

We used a Monte Carlo test to examine whether the potential penta-, hexa-, and heptapeptide targets were present among the genuine targets of T cell response significantly more often than random sets of SARS-CoV-2 penta-, hexa- and heptapeptides. We compared the representation of potential penta-, hexa- and heptapeptide targets in the genuine targets of T cell response with the representation of 983 pentapeptides, 7091 hexapeptides, and 9334 heptapeptides 10 000 times randomly selected from the SARS-CoV-2 proteome (Fig. 1). The results showed that our *in silico*-identified hexapeptides, but not pentapeptides or heptapeptides, were overrepresented in the empirically identified targets of T cell recognition in post-COVID patients (hexapeptides: genuine peptides 2124, random peptides 2186.03, *P* = 0.027, Cohen’s *d* = 1.97; pentapeptides: genuine peptides 296, random peptides 309.95, *P* = 0.834, Cohen’s *d* = 1.02; heptapeptides: genuine peptides 2525, random peptides 2526.49, *P* = 0.658, Cohen’s *d* = 0.47) (Fig. 3D). *Post hoc* tests performed separately for targets of cytotoxic and helper T cells showed that the results were significant for hexapeptides recognized by cytotoxic T cells (*P* = 0.040, Cohen’s *d* = 1.77, genuine 1159 versus random 1130.33) but not for those recognized by helper T cells (*P* = 0.075, Cohen’s *d* = 1.44, genuine 1457 versus random 1433.07). The effects were predominantly non-significant and considerably weaker than those described in the previous section, but still remained very large according to Cohen’s classification.

Table 2 shows the results of Monte Carlo tests performed separately for the whole virus (the upper section), for its spike protein originating from the human-adapted virus (the middle section), and for the rest of the virus, that is, the 10 genes which originated from the horseshoe bat-adapted SARS-CoV-2 progenitor (the bottom section). Overrepresentation of potential hexapeptide targets of T cell recognition in peptides eliciting a T cell response was significant only for those immunogenic peptides originating in the spike protein which elicited a helper T cell response. Analyses of proteins originating from the other ten proteins showed an overrepresentation of potential heptapeptide targets of T cell recognition which were present in some peptides stimulating a T cell response.

**Table 2:** Prevalence of potential targets which were present in peptides eliciting T cell response within all potential targets of T cell recognition

	No. targets	Within genuine	Within genuine (%)	Within random	Within random (%)	SD random	*P*	Cohen’s *d*
T cells, whole virus								
5-Peptides	983	296	30.1	309.95	31.5	13.71	0.834	1.02
6-Peptides	7091	2124	30.0	2086.03	29.4	19.29	0.027	1.97
7-Peptides	9334	2525	27.1	2526.49	27.1	7.39	0.658	0.47
Th cells, whole virus								
5-Peptides	983	198	20.1	206.35	21.0	11.98	0.743	0.70
6-Peptides	7091	1457	20.5	1433.07	20.2	16.64	0.075	1.44
7-Peptides	9334	1798	19.3	1802.75	19.3	6.54	0.751	0.73
Tc cells, whole virus								
5-Peptides	983	168	17.1	183.2	18.6	11.45	0.906	1.33
6-Peptides	7091	1159	16.3	1130.33	15.9	15.92	0.040	1.77
7-Peptides	9334	1215	13.0	1226.34	13.1	5.56	0.969	2.01
T cells, spike protein								
5-Peptides	111	78	70.3	80.12	72.2	4.51	0.646	0.47
6-Peptides	942	663	70.4	650.05	69.0	7.22	0.032	1.79
7-Peptides	1240	795	64.1	795.67	64.2	2.44	0.515	0.27
Th cells, spike protein								
5-Peptides	111	65	58.6	61.16	55.1	5.03	0.198	0.76
6-Peptides	942	518	55.0	501.46	53.2	7.83	0.015	2.11
7-Peptides	1240	621	50.1	618.52	49.9	2.54	0.118	0.98
Tc cells, spike protein								
5-Peptides	111	42	37.8	49.33	44.4	5.02	0.915	1.46
6-Peptides	942	369	39.2	364.09	38.7	7.58	0.238	0.65
7-Peptides	1240	391	31.5	393.41	31.7	2.35	0.793	1.03
T cells, other genes								
5-Peptides	614	218	35.5	234.77	38.2	11.35	0.924	1.48
6-Peptides	4132	1462	35.4	1455.64	35.2	15.51	0.336	0.41
7-Peptides	5387	1730	32.1	1721.19	32.0	5.76	0.049	1.53
Th cells, other genes								
5-Peptides	614	133	21.7	146.44	23.9	9.98	0.902	1.35
6-Peptides	4132	940	22.7	945.9	22.9	13.72	0.653	0.043
7-Peptides	5387	1177	21.8	1170.15	21.7	5.08	0.071	1.35
Tc cells, other genes								
5-Peptides	614	126	20.5	136.14	22.2	9.81	0.837	1.03
6-Peptides	4132	790	19.1	776.19	18.8	12.63	0.129	1.09
7-Peptides	5387	824	15.3	816.38	15.2	4.45	0.029	1.71

This table presents the total number of potential T cell recognition targets (Column 2, *No. targets*), the count of those actually present in peptides eliciting T cell responses (Column 3, *Within genuine*), their proportion among all potential T cell recognition targets (Column 4, *Within genuine %*), and the results of the Monte Carlo test. Specifically, the last five columns display the mean number of randomly selected pseudotargets present in peptides eliciting a T cell response (Column 5, *Within random*), their proportion within all pseudotargets (Column 6, *Within random %*), the standard deviation of the number of pseudotargets present in peptides eliciting a T cell response (Column 7, *SD random*), the significance of the one-sided Monte Carlo test (Column 8, *P*), and a Cohen's d indicating the effect size, representing the difference between columns 4 and 6 (Column 9, *Cohen's d*).

## Discussion

Our *in silico* method predicted a subpopulation of pentapeptides and hexapeptides with the potential to induce a T cell response in humans. These peptides were overrepresented among those experimentally identified as T cell targets in post-COVID patients. The method has relatively high sensitivity and relatively low specificity. Therefore, the absence of a parasite’s peptide in the host is a strong indicator of its immunogenicity; however, only a fraction of such potentially immunogenic peptides are present in peptides that really elicit T cell response.

About 70% of potential targets of T cell recognition were absent in all peptides which had been recognized as targets of T cell response by immunological assays. Many of them were probably absent for trivial reasons, such as the corresponding peptides were not being tested in the Tarke *et al.* study or not binding to any variant of MHC proteins of the 99 post-COVID patients involved in the study. There are, however, at least two other, less trivial explanations. To fit in the groove of MHC proteins and to be kept there firmly enough, specific amino acids must surround the peptide recognized by a T cell. Moreover, a potential immune response target can become an actual immune response target only if it is present in the protein expressed intensively enough in the virus-infected cells. Tarke *et al.* showed that most peptides stimulating a T cell response were present in just 4 out of 22 proteins, most frequently in the spike protein. Potential targets of T cell recognition present in the weakly expressed or less immunogenic proteins therefore had a low chance of being detected in an empirical study. Our study confirmed that a strikingly higher fraction of predicted targets (70%) were observed in empirically detected T cell immunogenic peptides originating from the highly expressed and highly immunogenic spike protein than in the whole SARS-CoV-2 proteome (30%). This explanation thus seems to be the most probable of all the (non-exclusive) explanations mentioned just above. In this context, it is essential to bear in mind that the density of potential pentapeptide targets of T cell recognition was lower in the highly expressed, highly immunogenic spike protein than in other SARS-CoV-2 proteins. This difference in densities aligns with the results of an earlier study on pentapeptide and hexapeptide vocabularies of coronaviruses [7]. The study compared the vocabularies of 11 human and bat coronaviruses with those of 38 different mammal species. It revealed that the spike protein of SARS-CoV-2 had the highest similarity to humans (it had the fewest number of pentapeptides absent in human proteins), while other proteins had the greatest similarity to the horseshoe bat. This suggests that SARS-CoV-2, and not any other viruses except the closely related RatG-13, is a chimera. Its spike protein originated from a donor initially adapted to a human host (therefore lacking most pentapeptides absent in the human peptidome), while the rest of its proteome originated from another coronavirus species initially adapted to horseshoe bats. The same analysis for the more rapidly evolving hexapeptide vocabularies indicated that the donor of the spike gene was recently passaged in rats (SARS-CoV-2) and mice (RatG-13), while the donor of other genes passed through another animal used in virological labs, specifically the treeshrew [7]. In typical parasites of non-chimeric origin, the densities of T cell recognition targets will be similar in all parts of the proteome. Hence, the correlation between the immunogenicity of an individual protein and the density of its potential T cell recognition targets will probably be higher for such parasites than for SARS-CoV-2. The practical implication of our discovery is that even a protein with a low density of potential T cell targets can be a highly potent immunogen if it is expressed at sufficiently high levels and/or when it is located on the pathogen’s surface. The impact of this finding on the strategy for developing effective vaccine candidates is outlined below.

All peptides that elicited T cell response in post-COVID patients contained at least one predicted hexapeptide and heptapeptide target of immune recognition, and about one half of the immunogenic peptides contained at least one of the less numerous pentapeptide targets. Results of the Monte Carlo tests show that such overrepresentation is highly improbable and cannot happen by chance. In fact, peptides of none of the 10 000 randomly selected sets of peptides were similarly overrepresented in the 280 helper T cell epitopes. This stronger overrepresentation of hexapeptides than pentapeptides (Table 2) might seem paradoxical. Intuitively, we would expect the presence of a potential hexapeptide target to be necessarily associated with the existence of a pentapeptide target. This principle, however, applies only in the reverse direction: the presence of potential pentapeptides is necessarily associated with the presence of hexapeptide targets. A comparison of our lists of potential T cell recognition targets showed that most of the potential hexapeptide T cell targets (76.7%) contained only pentapeptides which are present in the human proteome. As a result, a significant fraction of potential hexapeptide targets contains no potential pentapeptide target.

Upon comparing the overrepresentation of pentapeptides and hexapeptides, which contain at least one potential T cell target, we found that those which elicited a T cell response tended to be pentapeptides. This, in conjunction with the notably lower density of potential pentapeptide T cell targets in the spike protein (as compared to a less dramatic reduction in hexapeptides), suggests that T cells might predominantly target pentapeptides rather than hexapeptides. In contrast, the opposite was suggested by the analyses of overrepresentation of potential targets of recognition which are present in peptides that elicited T cell response within all potential targets of T cell recognition (Cohen’s *d* 1.02 versus 1.97). Earlier proteomic analyses indicated that pentapeptides, and not hexapeptides, are the primary targets of the T cell recognition. For example, a comparison of sizes of peptide vocabularies of 38 parasitic and 33 nonparasitic species showed that parasites of vertebrate hosts have impoverished pentapeptide vocabulary but enriched hexapeptide vocabulary [5]. Similarly, the relative genetic distance between the horseshoe bat and SARS-CoV-2 vocabularies, as well as between the human and SARS-CoV-2 spike protein vocabulary, was smaller for pentapeptides than for hexapeptides [7].

These discrepancies between previous and current results could be explained as follows: pentapeptides are recognized by T cell receptors (TCRs), but longer peptides are needed for attachment in the MHC protein groove [13]. The specificity of the bond between the MHC protein and the neighboring amino acids of the T-cell-recognized pentapeptide might be relatively low. This could be due to the possibility that several different amino acids (although certainly not all) surrounding a certain T-cell-recognized pentapeptide can facilitate its binding to a specific variant of the MHC protein. If a clone of T cell recognizes a particular pentapeptide, then probably several peptides containing different hexapeptides which include this pentapeptide are also recognized as immunogenic in immunological assays. It is possible that due to these “pseudoreplications”, statistical and Monte Carlo tests can more easily detect the enrichment of immunogenic peptides by hexapeptide targets than by pentapeptide targets. Due to this effect, we would more easily detect hexapeptides as targets of T cell recognition even if pentapeptides were the genuine targets of T cell recognition.

Regardless of whether pentapeptides or hexapeptides are being recognized by the T cells, the impoverishment of peptide vocabularies can be more easily recognized on the level of pentapeptides than on the level of hexapeptides. As discussed earlier [5], there are at least two processes that most likely affect the size of hexapeptide vocabularies. The first is the elimination of hexapeptides. This process necessarily accompanies the elimination of pentapeptides, because elimination of a pentapeptide can theoretically result in the elimination of as many as 40 different hexapeptides containing that particular pentapeptide. As argued earlier, the rate of elimination of hexapeptides from proteins during the parasite’s adaptation to a new host is probably faster than the rate of elimination of pentapeptides [7]. Another process that runs in the opposite direction is the enrichment of the hexapeptide vocabulary, aiming to compensate for the shortage in the number of pentapeptides. To build up biologically active proteins using a smaller collection of (pentapeptide) building blocks, the parasites must use these blocks more inventively—and this automatically results in a richer hexapeptide vocabulary.

The fact that many potential T cell targets were not detected in any immunogenic peptides can be explained easily, as discussed above. More interesting is the existence of about one-half of immunogenic peptides that do not contain any potential pentapeptide T cell targets. As discussed in the previous paragraphs, it is possible that hexapeptides, rather than pentapeptides—or, even more probably, both hexapeptides and pentapeptides—are the genuine targets of T cell recognition. It can be argued that experimental approaches (such as crystallography, site-directed mutagenesis, and binding inhibition experiments), rather than bioinformatics, can tell us whether pentapeptides, hexapeptides or both are recognized by T cells. Such experimental studies have already identified the amino acids which are in physical contact with the hypervariable CDR3 loop of TCR, as well as those which are necessary for the binding or correct positioning of a peptide in the groove of MHC molecules for a large number of MHC–peptide–TCR complexes [14]. Even these direct methods, however, are not omnipotent. Not all identified amino acids are necessarily part of the hexapeptide or pentapeptide that allowed T cell recognition. To repeat: the presence of a parasite’s peptide that is absent in the host vocabulary is a necessary but not sufficient condition of T cell recognition.

Another possible explanation for the total absence of predicted targets in some immunogenic peptides is that human T cells recognize even some pentapeptides of human origin as non-self. Elimination of potentially autoreactive T cells in the process of negative selection during their maturation does not work with a 100% efficiency. If some pentapeptides are present in only weakly expressed human proteins, or in proteins weakly expressed in the cells of the human thymus, then the corresponding T cells might survive the passage through the thymus. Consequently, these peptides may elicit a T cell response when they are present in sufficiently expressed viral proteins. This mechanism can play a role in some types of autoimmune disorders, because infection by a parasite can result in an expansion of initially rare potentially autoreactive T cell clones [15] and the existence of certain classes of regulatory T cells might be part of adaptation of the immune system to such situations.

It is also probable that, due to polymorphism in most human proteins, some pentapeptides are missing in the protein vocabulary of some individuals, including the individual whose proteome was used for the construction of peptide vocabularies in the present study (F _000001405.39). In future studies, or when designing vaccines, it might be helpful to prepare a more representative human peptide vocabulary by using the proteomes of several individuals of different ethnic origins. In fact, one could even construct different vocabularies used for the identification of potential T cell recognition targets for people of different geographic or ethnic origins. Such approaches could mitigate the effect of genetic polymorphism in human protein-coding genes.

An efficient vaccine must contain not only the antigen that could bind to B cell receptors (to membrane-bound and soluble immunoglobulins) but also peptides that could bind to receptors of helper T cells. Current algorithms for predicting which protein will elicit a T cell response are based on rules derived from empirical studies [16–18]. These algorithms yield valuable results when searching for peptides that stimulate helper T cell responses; however, they perform less effectively in identifying peptides that stimulate cytotoxic T cell responses. For instance, in the study by Tarke *et al*., 280 (14.3%) of all 1952 predicted epitopes induced helper T cell responses, while only 454 (8.1%) of all 5600 predicted epitopes induced cytotoxic T cell responses. In comparison, 30% of the 983 pentapeptides identified as potentially immunogenic by our method were present in peptides empirically found to elicit T cell responses. For those originating from the spike protein, as much as 70% were present in peptides that elicited the T cell response.

Our method for predicting potentially immunogenic peptides relies on rules derived from a theory, which is based on our understanding of the universal mechanism that allows the vertebrate adaptive immune system to distinguish between self and non-self. While our method demonstrates excellent sensitivity, its specificity is not as strong. Therefore, we recommend using our theory-based method when efficient empirically based algorithms are unavailable, such as in the development of some veterinary vaccines. In all other cases, we advise combining our method with empirically based approaches. In these situations, the optimal workflow for identifying immunogenic peptides (the first step in vaccine development) consists of using our method to find a set of viral peptides not present in the host’s proteome, and then employing empirically based algorithms to identify a subset that binds to the host’s MHC proteins. As demonstrated by the example of the spike protein, it is particularly advantageous to prioritize empirical testing of immunogenicity for peptides within this subset that are derived from either highly expressed proteins or surface proteins of the pathogen.

### Limitations

In the present study, we applied our method to the entire viral proteome. However, we only have information about the genuine immunogenicity for the subset of peptides that previously passed the empirically based algorithm screening and were therefore experimentally tested for immunogenicity by Tarke *et al*. [8]. It is highly possible that many peptides identified as potential targets of T cell response by our method are part of immunogenic peptides that were not tested in Tarke *et al.*’s study. Consequently, it is likely that the specificity of the new method is much higher than it appears based on our results. Future studies should compare the sensitivity and specificity of the new and old methods by testing the real immunogenicity of three subsets of peptides, namely peptides identified by the new method, the old method, and those identified simultaneously by both methods. Such studies, however, must be conducted in immunological laboratories, rather than in institutions focusing on theoretical biology.

## Conclusion

Our study showed that a new bioinformatic method based on comparing parasite and host peptide vocabularies can predict which peptides will be the targets of T cell recognition. This method is based on a different principle than the currently used predictive algorithms. It has correctly predicted 70% of immunogenic peptides in a strongly expressed and highly immunogenic spike protein of SARS-CoV-2. Therefore, this inexpensive and rapid method could most likely be deployed in designing new vaccines, particularly in the search for peptides likely to elicit helper and cytotoxic T cell responses in both human and veterinary medicine.

## Data Availability

All scripts are available at Figshare and GitHub. https://doi.org/10.6084/m9.figshare.17711474.v3, https://doi.org/10.6084/m9.figshare.20981719.v1 (https://github.com/jflegr/peptides).
